# Localization of HIV-1 Vpr to the nuclear envelope: Impact on Vpr functions and virus replication in macrophages

**DOI:** 10.1186/1742-4690-4-84

**Published:** 2007-11-26

**Authors:** Guillaume Jacquot, Erwann Le Rouzic, Annie David, Julie Mazzolini, Jérôme Bouchet, Serge Bouaziz, Florence Niedergang, Gianfranco Pancino, Serge Benichou

**Affiliations:** 1 Institut Cochin, Université Paris Descartes, CNRS (UMR 8104), Paris, France; 2 Inserm U567, Paris, France; 3 Unité de Régulation des Infections Rétrovirales, Institut Pasteur, Paris, France; 4 Inserm U640, CNRS UMR 8151, Paris, France

## Abstract

**Background:**

HIV-1 Vpr is a dynamic protein that primarily localizes in the nucleus, but a significant fraction is concentrated at the nuclear envelope (NE), supporting an interaction between Vpr and components of the nuclear pore complex, including the nucleoporin hCG1. In the present study, we have explored the contribution of Vpr accumulation at the NE to the Vpr functions, including G2-arrest and pro-apoptotic activities, and virus replication in primary macrophages.

**Results:**

In order to define the functional role of Vpr localization at the NE, we have characterized a set of single-point Vpr mutants, and selected two new mutants with substitutions within the first α-helix of the protein, Vpr-L23F and Vpr-K27M, that failed to associate with hCG1, but were still able to interact with other known relevant host partners of Vpr. In mammalian cells, these mutants failed to localize at the NE resulting in a diffuse nucleocytoplasmic distribution both in HeLa cells and in primary human monocyte-derived macrophages. Other mutants with substitutions in the first α-helix (Vpr-A30L and Vpr-F34I) were similarly distributed between the nucleus and cytoplasm, demonstrating that this helix contains the determinants required for localization of Vpr at the NE. All these mutations also impaired the Vpr-mediated G2-arrest of the cell cycle and the subsequent cell death induction, indicating a functional link between these activities and the Vpr accumulation at the NE. However, this localization is not sufficient, since mutations within the C-terminal basic region of Vpr (Vpr-R80A and Vpr-R90K), disrupted the G2-arrest and apoptotic activities without altering NE localization. Finally, the replication of the Vpr-L23F and Vpr-K27M hCG1-binding deficient mutant viruses was also affected in primary macrophages from some but not all donors.

**Conclusion:**

These results indicate that the targeting of Vpr to the nuclear pore complex may constitute an early step toward Vpr-induced G2-arrest and subsequent apoptosis; they also suggest that Vpr targeting to the nuclear pore complex is not absolutely required, but can improve HIV-1 replication in macrophages.

## Background

In contrast to oncoretroviruses that replicate only in dividing cells and require nuclear envelope (NE) disassembly during mitosis to integrate their genetic material into the host cell genome, HIV-1 and other lentiviruses have the ability to productively infect non-dividing cells, such as terminally-differentiated macrophages [1]. In the case of HIV-1, these cell populations represent important targets during the initial steps of infection and largely contribute to the establishment of viral reservoirs [2]. The ability of HIV-1 to infect non-dividing cells relies on mechanisms allowing active transport of the so-called "preintegration complex" (PIC), the nucleoprotein complex containing the viral DNA, from the cytoplasm to the nuclear compartment through the intact NE. While nuclear import of the PIC is essential for virus replication in non-dividing cells, it was also proposed that uncoating of the viral capsid after virus entry might rather be the rate-limiting step in the ability of HIV-1 to infect such non-dividing cells [3]. The molecular details underlying this process are still unknown, but a certain body of evidence suggests that the PIC may be transported along the microtubule network to accumulate at the nuclear periphery before anchoring to the NE (for review, see Ref. [4]).

Although the composition of the HIV-1 PIC changes during its travel to the nucleus, three viral proteins, namely the matrix protein (MA), integrase (IN) and the auxiliary viral protein R (Vpr), remain tightly associated with the viral DNA and have thus been proposed as potential mediators of the nuclear import of the PIC. The central DNA flap structure generated upon completion of the reverse transcription process has been involved in this active process. While the exact contribution of these distinct viral determinants in the nuclear import of the PIC is still controversial (for review, see Ref. [4]), HIV-1 Vpr specifically facilitates virus replication in non-dividing cells and differentiated macrophages [5-8]. In addition, it was recently reported that some tRNA species incorporated into virus particles may also promote nuclear import of the viral DNA [9].

HIV-1 Vpr is a highly conserved 96-amino acid (a.a.) basic protein (14 kDa). The analysis of the soluble full length Vpr polypeptide by nuclear magnetic resonance (NMR) allowed the three-dimensional (3D) structure determination of the protein. Vpr consists of an hydrophobic central core domain, with three α-helices (H1 a.a. 17–33, H2 a.a. 38–50 and H3 a.a. 55–77), that are connected by loops and surrounded by two flexible N- and C-terminal domains negatively and positively charged, respectively [10]. By contrast with other HIV-1 auxiliary proteins, Vpr is specifically incorporated at a high copy number in virus particles [11-15], and is consequently present in the cytoplasm of newly infected cells, indicating that it certainly plays specific roles in the early post-entry steps of viral replication [16]. In addition to its role in the nuclear import of the viral PIC, Vpr displays several other activities, including an effect on the fidelity of the reverse-transcription process, an arrest of the cell cycle at the G2/M transition, an induction of apoptosis and the transactivation of the HIV-1 LTR as well as host cell genes (for review, see Ref. [17]. Although the exact contribution of these activities along the virus life cycle is still debated, Vpr-induced G2-arrest has been proposed to provide a favorable cellular environment for optimal transcription of HIV-1 [18], while the modulation of the virus mutation rate seems required for efficient spreading of HIV-1 in primary macrophages [19].

When expressed either in dividing or non-dividing cells, HIV-1 Vpr displays evident karyophilic properties and is clearly concentrated at the NE at steady state [20-23]. This latter observation was correlated with its binding to several components of the nuclear pore complex (NPC) which selectively regulates the trafficking of macromolecules or complexes between the nucleus and cytoplasm [24-26]. The NPC is a large supramolecular structure embedded into the NE and composed of around 30 unique proteins termed nucleoporins (Nups) [27]. About half of these Nups contain Phe-Gly repeats (FG-repeats) that contribute directly to the active nucleo-cytoplasmic transport. While initial studies supported the idea that Vpr could bind the FG-rich regions of several Nups, including the human Nup54 and Nup58 [24], the rodent Pom121 [26] and the yeast Nsp1p [25], a more recent study described a direct interaction between Vpr and the human CG1 nucleoporin [28]. This interaction does not require the FG-rich region of hCG1 but rather a region without consensus motif found in the N-terminal domain of the protein. Using an *in vitro *nuclear import assay, it has been demonstrated that hCG1 contributed in the accumulation of Vpr to the NE [28].

Only a few reports have tried so far to evaluate the virological impact related to the property of HIV-1 Vpr to localize at the NE [25,29]. In the present study, we have explored the role of Vpr accumulation at the NE for the Vpr functions, including G2-arrest and pro-apoptotic activities, and for virus replication in primary macrophages. Single-point Vpr mutants, including two new independent mutants that specifically failed to interact with hCG1, were characterized. Like other mutants with substitutions within the first α-helix of Vpr, they failed to localize at the NE and were impaired for G2-arrest and cell death induction, indicating a functional link between these activities and the Vpr accumulation at the NE. Finally, the replication of the hCG1-binding deficient Vpr mutant viruses was impaired in monocyte-derived macrophages (MDMs) from some but not all donors, suggesting that Vpr targeting to the nuclear pore complex is not absolutely required, but can improve HIV-1 replication in macrophages.

## Results

### Identification of Vpr mutants deficient for hCG1-binding

**Figure 1:**
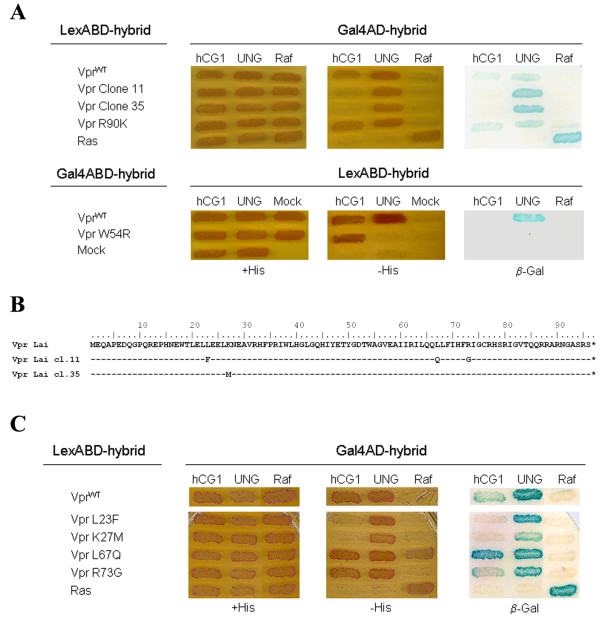
**Identification of Vpr mutants deficient for binding to the nucleoporin hCG1**. A) Screening for Vpr mutants defective for the interaction with hCG1. The L40 yeast reporter strain expressing the wt or mutated (clones 11 and 35, and Vpr-R90K and -W54R single-point mutants) HIV-1 Vpr fused either to LexABD (upper panels) or to the Gal4 DNA binding domain (Gal4BD) (lower panels), in combination with each of the Gal4AD-hybrids indicated on the top was analyzed for histidine auxotrophy and β-Gal activity. Double transformants were patched on selective medium with histidine (+His) and then replica-plated on medium without histidine (-His) and on Whatman filters for β-Gal assay. Growth in the absence of histidine and expression of β-galactosidase indicated an interaction between hybrid proteins. B) Amino acid substitutions found in the hCG1-binding deficient Vpr mutants (clones 11 and 35). Mutants were derived by error prone PCR-mediated mutagenesis from the primary sequence of the VprLai strain that is shown at the top. C) Isolation of single-point Vpr mutants defective for the interaction with hCG1. Single-point mutants derived from Vpr clones 11 and 35 fused to LexABD were expressed in L40 strain in combination with each of the Gal4AD-hybrids indicated on the top. Double transformants were assessed as described in A).

Previous studies have established that the localization of HIV-1 Vpr to the NE is related to its ability to interact with components of the NPC [23,25,26], including the nucleoporin hCG1 [28]. In order to identify single-point mutations that altered the Vpr binding to hCG1, we generated a library of random Vpr mutants and used the yeast two-hybrid system to screen for hCG1-binding deficient Vpr mutants. Only mutants which retained the capacity to interact with UNG2 and HHR23A, two other known relevant host partners of Vpr [30,31] but failed to bind hCG1 were selected. Two Vpr mutants (clones 11 and 35) that still interacted with UNG2 and HHR23A were isolated (Fig. 1A and data not shown, respectively), as evidenced by growth of yeast-transformed cells on medium without histidine (-His) and β-gal activity. In contrast, these mutants did not bind to hCG1, since no growth on -His medium and β-gal activity was observed. Used as controls, the VprR90K mutant, which is known to abolish Vpr-induced G2-arrest [31], still bound both to hCG1 and UNG2, while the W54R mutant, which is deficient for binding to UNG2 [32], still interacted with hCG1 (Fig. 1A, lower panel). These results show that this yeast two-hybrid strategy is a powerful system to isolate specific hCG1-binding deficient Vpr mutants.

DNA Sequencing of clone 11 revealed 3 substitutions within the VprLai primary sequence (Leu23Phe, Leu67Gln and Arg73Gly), while clone 35 contained a single substitution (Lys27Met) (Fig. 1B). Each substitution from clone 11 was introduced in the Vpr sequence and the 3 single-point mutants were analyzed again for binding to hCG1 and UNG2. As shown in Fig. 1C, the L23F and K27M substitutions were sufficient to abrogate hCG1 binding without significant alteration of binding to UNG2. In contrast, the L67Q and R73G Vpr mutants still interacted with both hCG1 and UNG2. These results reveal that the L23F and K27M Vpr variants are specifically altered for the binding to hCG1.

**Figure 2:**
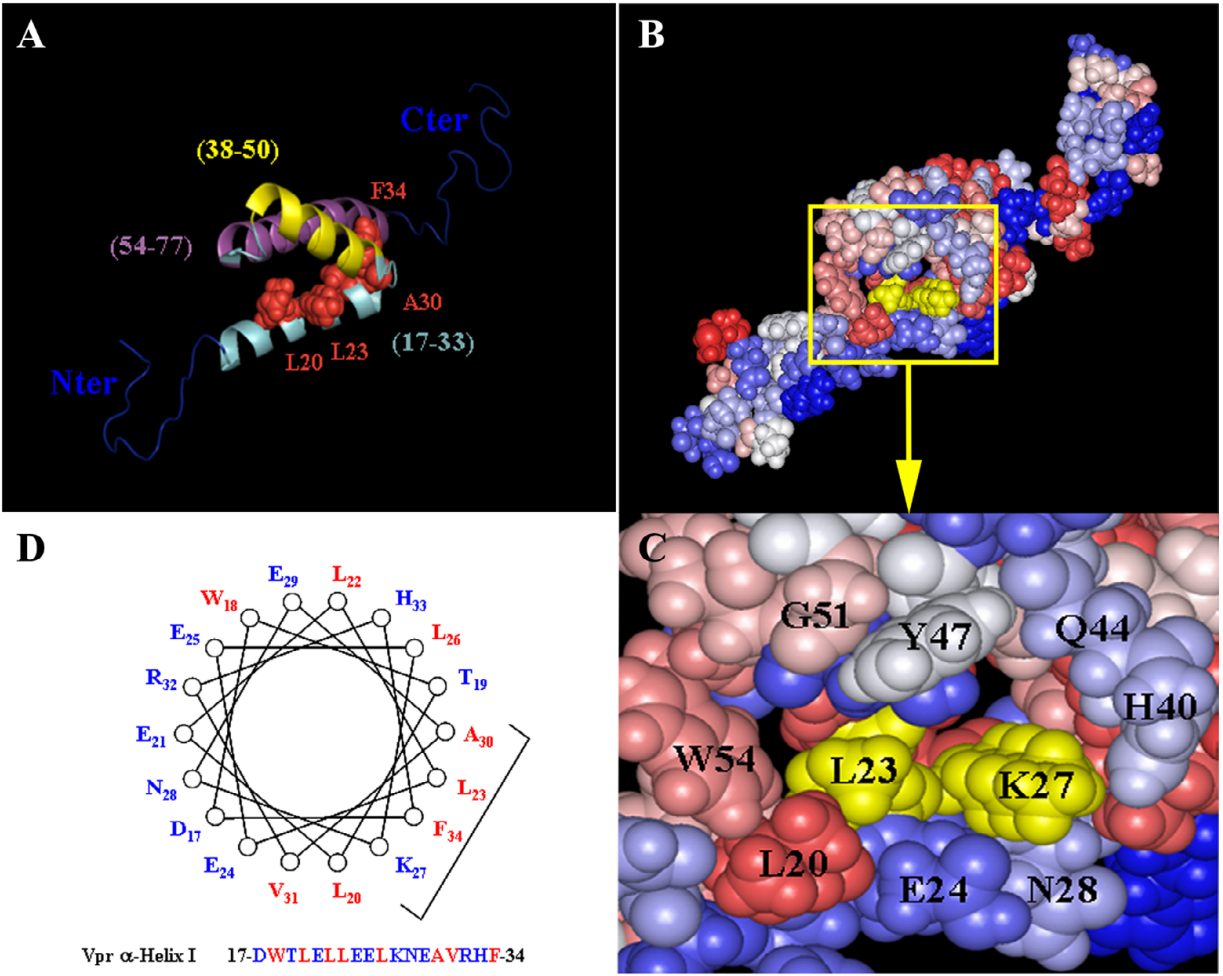
**Impact of the Vpr-L23F and -K27M substitutions on the three-dimensional structure of Vpr**. A) 3D structure of HIV-1 Vpr [10], showing the three α-helices (residues 17–33, 38–50 and 54–77) represented in light blue, yellow and purple, respectively. The L23, K27, A30 and F34 residues are colored in red. The unstructured N- and C-terminal domains are represented in dark blue. B) CPK representation of Vpr. Residues are colored according to their hydrophobicity, except for L23 and K27 which are colored in yellow. The yellow box is enlarged in C), and this region shows a pocket that is organized around the L23 and K27 residues within the first α-helix and may represent a site for hCG1 binding. D) Helical-wheel diagram of the first α-helix of Vpr extending from a.a. D17 to F34. Residues L23, K27, A30 and F34 which have been mutated in the present study are indicated. Hydrophilic residues are in blue, whereas hydrophobic residues are in red.

As deduced from the 3D structure organization of Vpr resolved by NMR (see on Fig. 2A), the Leu23 and Lys27 residues are located in the first N-terminal α-helix H1 (residues 17–33) of Vpr which has amphipathic properties. Leu23 and Lys27 are separated by 3 residues and are thus located on the same face of the first α-helix (Fig. 2D). The connection between these two residues is favored by the formation of a hydrogen-bonding network through the O19/NH23, O23/NH27 and O27/NH31 atoms maintaining the structure of the α-helix. Moreover, the Corey, Pauling, and Koltun (CPK) representation, indicates that the Leu23 and Lys27 residues are located at the bottom of a pocket that is easily accessible to the solvent (Fig. 2B) and could constitute a binding site for hCG1. In addition, the Leu23 residue is hydrophobic and is surrounded by rather hydrophobic residues (Leu20, Trp54, Gly51 and Tyr47) that border one edge of the pocket (Fig. 2C), whereas the Lys27 residue is hydrophilic, positively charged and bordered by hydrophilic residues (Gln44, His40, Asn28 and Glu24) that constitute the second edge of the pocket. The potential structural modifications induced by substitution of Leu23 and Lys27 in Phe and Met, respectively, have been calculated by homology with the wild type Vpr protein using the Swiss-Model program [33-35]. The analysis indicated that the structure of the first α-helix (residues 17–33) is conserved as well as the hydrogen-bonding network allowing the stabilization of the 3 helices of HIV-1 Vpr. This supports the notion that the global 3D structure of the protein is not modified in these two Vpr mutants, as suggested from the yeast two-hybrid analysis.

### Intracellular distribution of the Vpr mutants

**Figure 3:**
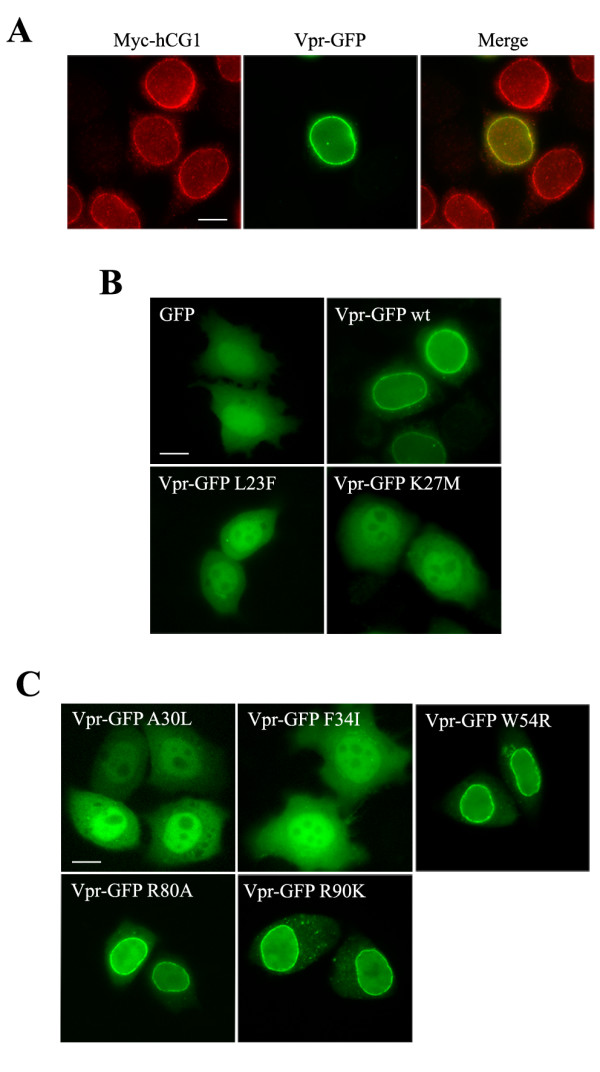
**Subcellular distribution of the Vpr mutants**. A) Colocalization of Vpr and hCG1 at the NE. HeLa cells co-expressing Vpr-GFP (middle row) and Myc-hCG1 (left row) fusion proteins were permeabilized with digitonin, fixed, and subsequently stained with an anti-Myc monoclonal antibody. B and C) Localization of wt and mutated Vpr-GFP fusions. HeLa cells expressing either GFP, wt Vpr-GFP, or the indicated Vpr-GFP mutants were fixed and directly examined. Cells were analyzed by epifluorescence microscopy, and images were acquired using a CCD camera. Scale bar, 10 μm.

Since HIV-1 Vpr localizes predominantly in the nucleus but also concentrates at the NE as a nuclear rim staining (Fig. 3A, middle panel) where it co-localizes with the nucleoporin hCG1 (left panel) [28], the cellular distribution of the two hCG1-binding deficient Vpr mutants was first analyzed. In contrast to the wt Vpr-GFP fusion, both Vpr-L23F and -K27M equally distributed between the cytoplasm and the nucleus (Fig. 3B), but they were excluded from the nucleolus. When expressed as HA-tagged proteins, these Vpr mutants similarly co-distributed in the cytoplasm and the nucleus, whereas wt HA-Vpr was concentrated into the nucleus and at the NE (data not shown). These data support that mutations of Vpr which alter its binding to hCG1 also impair its accumulation at the NE.

In order to explore whether substitutions in the first α-helix had a general impact on the localization of Vpr, the cellular distribution of two other Vpr mutants (Vpr-A30L and -F34I) was also analyzed (Fig. 3C). In contrast with published observations [36], we found that Vpr-A30L was distributed between the nucleus and the cytoplasm and failed to concentrated at the NE. As previously reported [25], Vpr-F34I displayed a nucleocytoplasmic distribution. In contrast, other Vpr mutants with substitutions in the third α-helix or in the C-terminal flexible basic region of the protein, such as Vpr-W54R, -R80A and -R90K, were concentrated at the NE as efficiently as the wt Vpr-GFP fusion (Fig. 3C). Altogether, these results indicate that the first α-helix of Vpr contains the major determinants required for the nuclear localization of the protein.

### G2-arrest activity and cell death induction of the Vpr mutants

**Figure 4:**
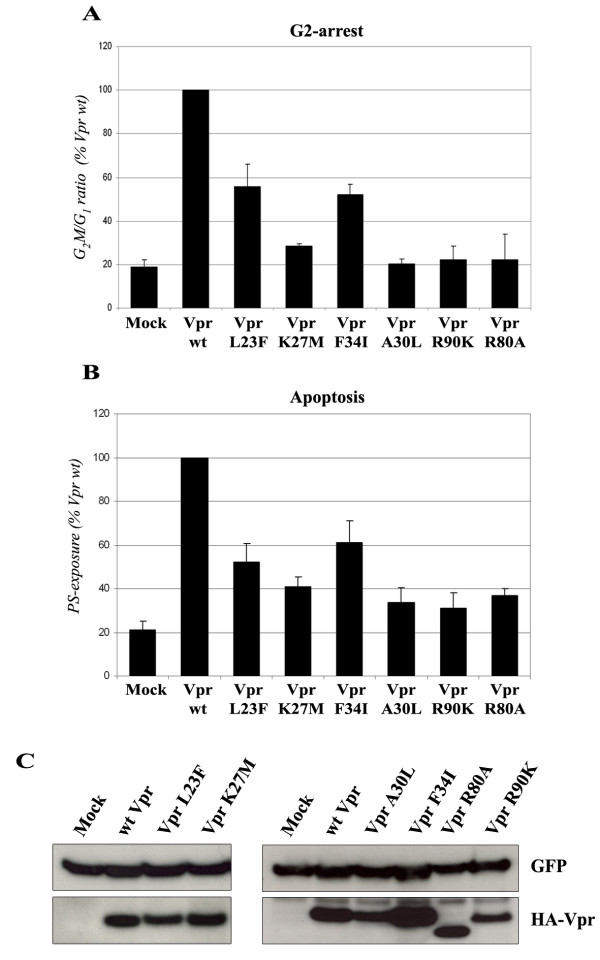
**G2-arrest and pro-apoptotic activities of the Vpr mutants**. HPB-ALL T cells were transfected with the HA-tagged Vpr (wt or mutated) expression vectors in combination with the GFP expression vector. A) G2-arrest activity. The DNA content was analyzed 48 h after transfection by flow cytometry on GFP-positive cells after staining with propidium iodide. Results are expressed as the percentage of the G_2_M/G1 ratio relative to that of the wt HA-Vpr. Values are the means of four independent experiments. Error bars represent 1 standard deviation from the mean. B) Pro-apoptotic activity. Cell surface PS exposure was analyzed 72 h after transfection by flow cytometry on GFP-positive cells after staining with phycoerythrin-labelled Annexin V. Results are expressed as the percentage of GFP-positive cells displaying surface PS exposure relative to that measured with wt HA-Vpr. Values are the means of four independent experiments. Error bars represent 1 standard deviation from the mean. C) Expression of wt and mutated HA-tagged Vpr proteins. Lysates from HPB-ALL transfected cells were analyzed by western-blotting using anti-GFP (upper panels) and anti-HA antibodies (lower panels).

Since a functional link was reported between the targeting at the NE and the Vpr-induced cell cycle arrest [36,37], the G2-arrest activity of the Vpr-L23F and Vpr-K27M mutants was first assessed in T lymphocytes. HPB-ALL T lymphoid cells were transfected with wt or mutated HA-tagged Vpr expression vector together with a GFP expression vector (see Fig. 4C), and the DNA content was analyzed 48 h later by flow cytometry on GFP-positive cells after staining with propidium iodide. The results of four independent experiments are recapitulated on Fig. 4A. The Vpr-L23F mutant was affected but retained about 50% of the activity measured for the wt protein, while the Vpr-K27M mutant was more severely affected leading to a residual G2-arrest activity. Consistent with previous observations, the Vpr-F34I mutant was partially altered for the G2-arrest activity [25], while the Vpr-A30L mutant was completely defective [20,36] (Fig. 4A). As controls, the Vpr-R80A and -R90K variants, which still accumulated at the NE (Fig. 3C), were unable to induce a G2-arrest (Fig. 4A and Refs. [31,37]). The pro-apoptotic activity of the wt Vpr protein and the mutants was also assayed, 72 h after transfection, by flow cytometry analysis of the cell surface exposure of phosphatidylserine (PS) after staining with phycoerythrin-labeled Annexin V (Fig. 4B). Interestingly, the Vpr-induced pro-apoptotic activity of all the Vpr mutants, including Vpr-L23F and -K27M, strictly paralleled the results obtained in the cell cycle experiments (compare Fig. 4A and 4B), suggesting that induction of G2-arrest and apoptosis by HIV-1 Vpr are functionally related. As evidenced on Fig. 4C, the reduction in G2-arrest and cell death induction observed with the Vpr mutants could not be explained by important differences in their expression levels, since all mutants were correctly expressed in HPB-ALL T lymphoid cells.

Altogether, these observations indicate that accumulation of Vpr at the NE is required but is not sufficient for its action on the cell cycle progression and the subsequent cell death. They also confirm that these two Vpr functions are functionally related.

### Intracellular localization of Vpr mutants in primary human monocyte-derived macrophages

**Figure 5:**
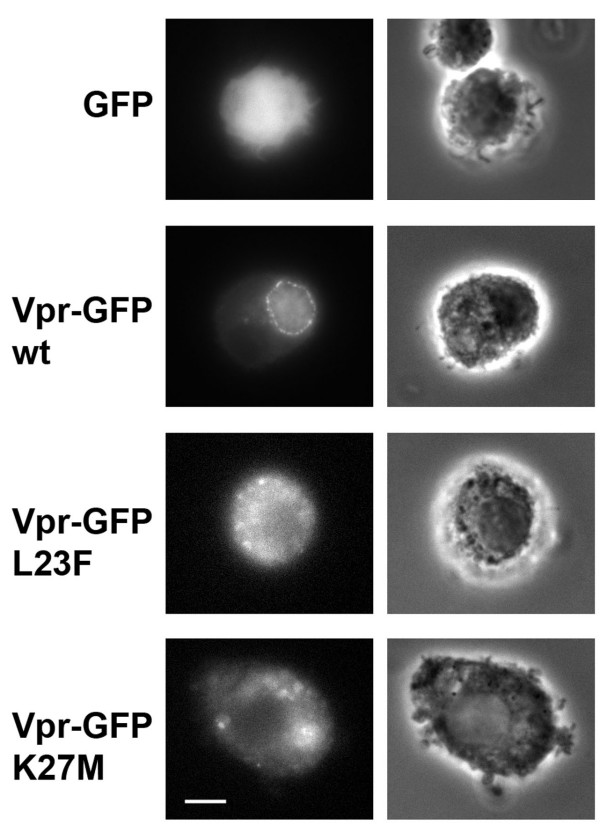
**Subcellular localization of wild type Vpr and Vpr mutants in human monocyte-derived-macrophages**. MDMs expressing either GFP, wt Vpr-GFP, or the indicated Vpr-GFP mutants were fixed and analyzed by wide-field microscopy. Z stacks of fluorescent images were acquired using a piezo with a 0.2 μm increment and one medial section is shown (left panels). Phase contrast images of the same cells were acquired to identify the nucleus (right panels). Scale bar, 5 μm.

In order to confirm that Vpr also accumulated at the nuclear envelope in target cells relevant for HIV-1 replication, the distribution of both wt and mutated Vpr proteins was then analyzed in primary macrophages derived from monocytes (MDMs) isolated from buffy coats of healthy donors. As previously shown in HeLa cells (see Fig. 3), the wt Vpr-GFP fusion localized in the nucleus of MDMs but also concentrated at the NE as a punctuate staining likely corresponding to NPC structures (Fig. 5). A similar punctuate staining at the NE was observed in a myeloid cell line, such as THP-1 cells, expressing the Vpr-GFP fusion (not shown). Again, both Vpr-L23F and -K27M mutants failed to concentrate at the NE and predominantly localized in the cytoplasm as a diffuse staining. These data confirm that Vpr mutants deficient for hCG1-binding also fail to accumulate at the NE in primary macrophages.

### Replication in primary macrophages of the hCG1-binding deficient Vpr HIV-1 mutants

**Figure 6:**
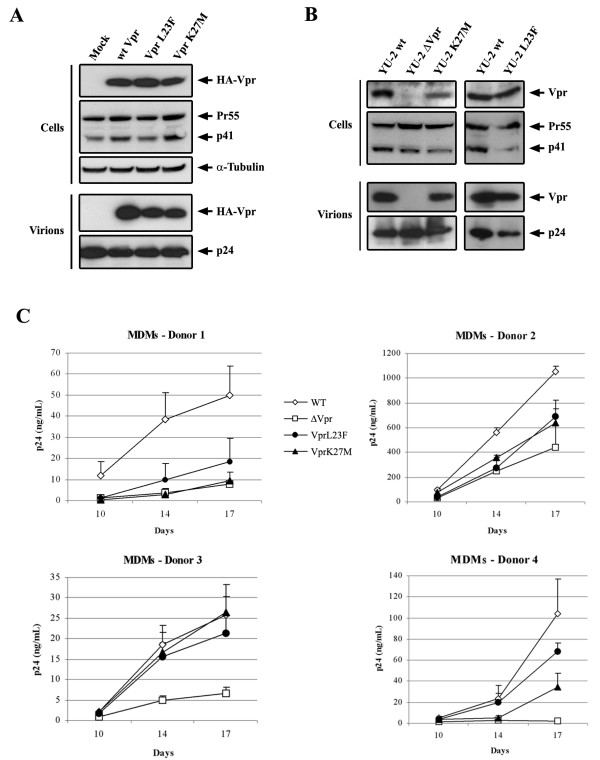
**Impact of the Vpr mutations on HIV-1 replication in monocyte-derived macrophages**. A) Packaging assay of the wt and mutated HA-tagged HIV-1 vpr into virus like particles. 293T cells were transfected with an HIV-1-based packaging vector lacking the *vpr *gene in combination with vectors for expression of the wt or mutated HA-tagged Vpr protein. 48 h later, proteins from cell and virion lysates were separated by SDS-PAGE and analyzed by Western blotting with anti-HA and anti-CAp24 antibodies. B and C) The L23F or K27M mutations were introduced into the *vpr *gene of the HIV-1YU-2 molecular clone. In B) Lysates from transfected 293T cells and virions isolated from cell supernatants were subjected to SDS-PAGE followed by Western blotting, using a rabbit polyclonal anti-Vpr and a mouse anti-CAp24 (provided from the NIH AIDS Research and Reference Reagent Program). In C) Replication of wild type and mutated HIV-1 in monocyte-derived macrophages. The wild type HIV-1YU-2 (WT, open diamonds) and the *vpr*-defective (ΔVpr, open squares), Vpr-L23F (black circles) and -K27M (black triangles) mutant viruses were produced by transfection of 293T cells with proviral DNAs. Monocyte-derived macrophages from four healthy donors were infected in triplicates with 0.5 ng of CAp24. Virus production was then monitored by measuring the p24 antigen by ELISA 10, 14 and 17 days after infection. Results are expressed as the level of p24 in the supernatants of infected cells. Values are the means of four experiments and error bars represent 1 standard deviation from the mean.

Finally, the relationship between the Vpr docking at the NE and HIV-1 replication in non-dividing cells was explored by analyzing the impact of the hCG1-binding deficient Vpr-L23F and -K27M mutations on viral replication in primary macrophages. The requirement of Vpr for early stages of the virus life cycle, including nuclear transport of the viral DNA (for review, see Ref. [17]), has been associated with its packaging into virions and the resultant presence in the cytoplasm of newly infected cells. Using a transient Vpr packaging assay in which HA-tagged Vpr is expressed in *trans *in virus producing cells [32], we therefore analyzed whether the two Vpr mutants were incorporated into virions. As evidenced in Fig. 6A, both Vpr-L23F and -K27M were efficiently packaged into purified virions, but a slight difference in the level of incorporation was repeatedly observed.

The L23F and K27M mutations were thus introduced into the *vpr *gene of the macrophage-tropic HIV-1YU-2 molecular clone. As a negative control, we used the isogenic *vpr*-defective mutant HIV-1YU-2ΔVpr, which contains two stop codons in frame without altering the *vif *open reading frame. As shown in Fig. 6B, both mutated VprYU-2 proteins were efficiently incorporated into purified HIV-1YU-2 virus particles, even if the slight difference in the level of incorporation of the two Vpr mutants evidenced in panel A was still apparent. We first verified that the Vpr mutant viruses did not show replication defects in cells permissive for *vpr*-defective virus replication *in vitro*. HeLa-CD4 cells or primary lymphocytes were first infected with equivalent inocula of wt or mutant viruses. Similar replication kinetics were observed for wt HIV-1YU-2, HIV-1ΔVpr, and the Vpr-L23F and -K27M mutant viruses (data not shown). We then infected monocyte-derived macrophages (MDMs) from 4 healthy seronegative donors with the same viral inocula. Consistent with previous reports [5-8], the *vpr*-defective virus showed a marked replication defect in MDMs from all donors (Fig. 5B). The Vpr-L23F and -K27M mutant viruses exhibited differential replication abilities according to the donor. Compared to the wt virus, a significant decrease of replication levels of the mutant viruses was observed in MDMs from three out of four donors (Fig. 5B, donors 1, 2 and 4). Conversely, the levels of replication of the Vpr-L23F and -K27M mutant viruses were similar to that of the wt virus in MDMs from another donor (Fig. 5B, donor 3). Although we cannot exclude that the replication defect observed in donors 1, 2 and 4 may be related to the differential virion incorporation evidenced in Fig. 5A, one can note that the levels of incorporation were sufficient, at least in donor 3, for efficient replication of the Vpr-L23F and -K27M mutant viruses. While these results confirm that the absence of Vpr expression consistently affects HIV-1 replication in primary macrophages, the Vpr-L23F and -K27M mutations lead to a replication defect in macrophages from most of the donors.

## Discussion

Although several studies have suggested a specific role for HIV-1 Vpr in facilitating the nuclear localization of the viral DNA during infection of non-dividing cells, such as macrophages, there is still no evident correlation reported in the literature between its real contribution to this process and the known functions of Vpr *in vitro*, including its ability to accumulate at the NE. Given that Vpr is dynamically associated with the NE [25,28], this subcellular distribution may be a pre-requirement for one or more of its known functions. Based on the findings that Vpr is able to interact directly with some proteins of the NPC, including the nucleoporin hCG1 [28], we have now identified two Vpr mutants, Vpr-L23F and -K27M, that are specifically deficient for hCG1-binding. Both mutations similarly abrogate Vpr concentration at the NE both in HeLa cells and in primary human monocyte-derived macrophages, supporting the hypothesis that this nucleoporin participates in the docking of the protein to the NPC. To our knowledge, it is the first report confirming that HIV-1 Vpr efficiently accumulates at the NE in primary macrophages. However, direct evidences regarding the specific role of hCG1 in the NE concentration of Vpr are still missing, since we failed so far to significantly deplete the endogenous hCG1 protein by using the RNA interference technology.

While substitutions of the Leu23 residue have been described previously [37,38], the mutation at position 27 (K27M) was not yet identified and is particularly interesting since the K27 is well-conserved among HIV-1 isolates and constitutes the only lysine residue along the whole Vpr sequence. This residue may potentially constitute a site for post-translation modifications, such as methylation, acetylation, hydroxylation, sumoylation or ubiquitination [39]. None of these modifications have been described previously for Vpr and our western blot analysis did not reveal any change in the level of expression and/or stability of the Vpr-K27M mutant compared to the wt protein. Interestingly, both Leu23 and Lys27 residues are located in the first N-terminal α-helix H1 (residues 17–33) of Vpr which has amphipathic properties (see on Fig. 2). The structural analysis confirms that the 3D structure and the stability of the three α-helices of Vpr are not significantly affected by the L23F and K27M substitutions, as indicated by the overall conservation of the hydrogen-bonding network of the protein. This analysis also reveals that the Leu23 and Lys27 residues are located in a close proximity at the end of the first α-helix, in a pocket that is easily accessible for protein-protein interaction with cellular partners, such as nucleoporins. We can notice that the two other mutations, A30L and F34I, impairing the NE accumulation of Vpr involve amino acids located on the same face of the first α-helix of the protein (see on Fig. 2D). However, Ala30 and Phe34 are not accessible and are rather involved in the stability of the structure by establishing hydrophobic interactions with residues of the third α-helix (55–77) of Vpr (see on Fig. 2A). Proximities between Ala30 and Leu64/Leu68 and between Phe34 and Leu64/Leu67/Leu68 have been identified from NMR experiments, indicating that Ala30 and Phe34 are directly involved in the interaction between the first and the third α-helix. Any mutation of the residues found at this interface will likely perturb the structure of the Vpr protein.

Our functional analysis raises intriguing questions concerning the functional link between Vpr accumulation at the NE and the *in vitro *properties of the protein, namely G2-arrest and cell death induction. Like other Vpr mutants that fail to localize at the NE, such as Vpr-A30L and -F34I, both L23F and K27M substitutions affect the Vpr-induced G2-arrest and cell death. These observations highlight two important points: i) the functional link between Vpr docking at the NE and its ability to cause a G2-arrest, and ii) the link between the G2-arrest and the pro-apoptotic activities of Vpr. First, it is possible that the accumulation at the NE could constitute a prerequisite for the Vpr-induced G2-arrest [40]. It was reported that Vpr provoked herniations and transient ruptures of the NE, resulting in a mixing of cytoplasmic and nuclear components that could contribute to the cell cycle arrest. Nonetheless, the molecular mechanism underlying this process is still unknown, and the local bursting caused by Vpr at the NE in this report has not been confirmed to date, even in imaging experiments performed on living cells [28]. Alternatively, the concentration of Vpr at the NE, or in a close vicinity of the nuclear pore complexes, could be required for the establishment of local interactions with some cellular partners involved in the regulation of the cell cycle. Because no mutant of HIV-1 Vpr has been described so far as disrupting the NE accumulation but keeping intact G2-arrest activity, our results confirm these observations and suggest that Vpr accumulation at the NE may be required. However, targeting to the NE is not sufficient for the G2-arrest activity, since several Vpr point mutants with substitutions in the C-terminal basic region of the protein, such as Vpr-R90K and Vpr-R80A, have been reported as defective for the G2-arrest activity (present study and Refs. [31,40,41]), while we show that both can still accumulate at the NE. Recent studies have shown that these residues may be involved in the direct recruitment of an unknown cellular factor that is required for G2-to-M transition [42-46].

Second, our results show that the pro-apoptotic activity of Vpr totally parallels the Vpr-induced G2-arrest, confirming previous reports suggesting that apoptosis is a direct consequence of the prolonged cell cycle arrest induced in Vpr-expressing cells [47-51]. Whereas other authors have suggested that these two Vpr properties were separated [22,41,52-54], it was recently demonstrated that the Vpr-induced apoptosis is directly dependent on the cell cycle arrest [55]. Both Vpr activities are directly related to the activation of G2 checkpoint ATR-initiated DNA damage-signaling pathway.

Though it is admitted that Vpr displays evident affinity for the NE [21-23,25,28], the biological significance of this property for efficient virus replication in non-dividing cells has been explored only in a few reports [25,29]. While a previous study showed that virus expressing single-point mutants (Vpr-F34I and -H71R), which failed to concentrate at the NE, displayed decreased infectivity in macrophages [25], it was recently reported that a Vpr mutant with multiple substitutions, in which the 4 Leu residues, including Leu23, found within the first α-helix of the protein were replaced by Ala, failed to localize in the nucleus and rendered the virus unable to replicate in MDMs from two donors [29]. Here, we show that the substitution of the Leu23 residue is sufficient to impair both nuclear accumulation of the Vpr protein and virus replication in primary macrophages. In addition, mutation of the Lys27 residue found in a close proximity within the first helix similarly impaired HIV-1 replication in MDMs from most donors. While the Vpr-L23F and -K27M mutant viruses displayed a wild-type level of replication in only one out of four donors, we cannot formally exclude that the replication defect observed in the three other donors may be related to the slight difference in the level of virion incorporation of the two Vpr mutants evidenced in Fig. 6A and 6B. As reported previously, the first α-helix of HIV-1 Vpr contains the main determinants required for efficient incorporation into virions [12,20,36,37].

Primary cells and especially MDMs are heterogeneous cell populations that greatly vary in their susceptibility to HIV infection [5,56]. Moreover, it is most likely that the contribution of Vpr for virus replication in macrophages relies on more than one of its activities. In addition to its role in nuclear import of the viral DNA, Vpr displays several other activities that could be important to maintain efficient virus replication in macrophages. Since it was reported that the manipulation of the cell cycle by Vpr increased viral expression in both dividing and non-dividing cells [18,57], the replication defect observed here with the mutant viruses could be, at least in part, related to the impairment of the Vpr-mediated G2-arrest. Additionally, the Vpr effect on the fidelity of the reverse-transcription process was also directly correlated with the ability of HIV-1 to efficiently replicate in primary macrophages [19]. These findings confirm that the absence of Vpr expression is deleterious for HIV-1 replication in primary macrophages, but additional analyses are required to re-evaluate the relationship between the multiple functions of Vpr characterized *in vitro *and its requirement *in vivo *for efficient replication in the non-dividing target cells of HIV-1.

## Conclusion

The present study shows that the targeting of Vpr to the nuclear pore complex may constitute an early step toward Vpr-induced G2-arrest and subsequent apoptosis. However, this localization at the NE is not absolutely required, but could improve HIV-1 replication in macrophages.

## Methods

### Expression plasmids and proviral DNAs

**Most of the **yeast and mammalian expression plasmids used in this study have been described previously [12,19,28,31], except plasmids for expression of the Vpr mutants with L23F and K27M substitutions; the Vpr mutant with the R80A substitution was kindly provided by E. Cohen (Universite de Montreal, Montreal, Canada). The L23F and K27M mutants were constructed by PCR-mediated site-directed mutagenesis using specific primers containing the desired mutations. The PCR product was then cloned between the *BamH*I-*Sal*I restriction sites of the pLex10 plasmid [12,31] and *BamH*I-*Xho*I of the pAS1b plasmid [28] to obtain vectors for expression of Vpr fused either to the LexA DNA binding domain (LexABD) or to the HA-tag, in yeast and in mammalian cells, respectively. For expression of Vpr mutants as N-terminal fusions with the green fluorescent protein (GFP), *vpr *mutated sequences were amplified by PCR with specific primers and cloned into pEGFP-N1 (Clontech).

Mutations of the *vpr *gene from the HIV-1 YU-2 molecular clone (obtained from the NIH AIDS Research & Reference Reagent Program) have been done using a shuttle vector containing the *Nde*I fragment (from nucleotide 5121 to 6401 according to HIVYU2X accession number) cloned into the pUC19 plasmid. Mutations (L23F, K27M) were thus performed using pUC19-VprYU-2 as a matrix by site-directed mutagenesis with specific primers: L23F-F ACACTAGAG CTTTTTGAGGAGCTTAAG, L23F-R CTTAAGCTCCTCAAAAAGCTCTAGTGT, K27M-F CTTTTAGAGGAGCTTATGAGAGAAGCTGTTAG, K27M-R CTAACAGCTTCTCTCATAA GCTCCTCTAAAAG. HIV-1YU-2Δ*vpr *has been constructed using the same procedure by insertion of two stop codons within the *vpr *gene without altering the *vif *gene using the following set of primers: Forward GATAGATGGAATAAGCCCCAGAAGACTAAGGGCCACAGAGG; Reverse CCTCTGTGGCCCTTAGTCTTCTGGGGCTTATTCCATCTATC. All constructs were verified by nucleotide sequencing with a DYEnamic ET Terminator kit (Amersham) and a Genetic Analyzer (ABI3100 Applied Biosystems).

### Yeast two-hybrid assay

The library of HIV-1 Vpr mutants fused to LexABD and the two-hybrid screening procedure of the library have been described [12,31]. Briefly, the *vpr *gene from the HIV-1 Lai isolate was amplified with error-prone Taq DNA polymerase (Promega Inc.) using previously described conditions [31,58], and the fragments were then inserted between *BamH*I and *Sal*I sites of pLex10. Library plasmid from about 10^5 ^independent *E. coli *clones, representing the complexity of the *vpr *mutant library, was prepared and used to transform the L40-MATα yeast strain. About 10^5 ^yeast clones were then screened to select Vpr mutants defective for binding to hCG1, by mating with the AMR70-MATα yeast strain previously transformed with the Gal4AD-hCG1 expression vector [28]. The yeast clones were also mated with the AMR70 strain previously transformed with the Gal4AD-UNG2 expression vector [19]. Plasmids from two mutants unable to interact with hCG1, but still binding to UNG2, were rescued and their insert was completely sequenced. Since one variant contained several point mutations (L23F, L67Q, and R73G; see on Fig. 1B), the single-point variants were constructed by site-directed mutagenesis. The L40 yeast reporter strain containing the two LexA-inducible genes, *HIS3 *and *LacZ*, was cotransformed with the indicated LexABD and Gal4AD hybrid expression vectors and plated on selective medium lacking tryptophan and leucine as previously reported [12]. Double transformants were patched on the same medium and replica-plated on selective medium lacking tryptophan, leucine, and histidine for auxotrophy analysis, and on Whatman 40 filters for β-galactosidase (β-gal) activity assay [12]. This latter assay was monitored by incubation for 1 h to 4 h at 30°C, and the reaction was then stopped with 1 M Na2CO3.

### Cell culture and transfections

HeLa cells were maintained in Dulbecco's modified Eagle's medium (DMEM, Invitrogen) supplemented with 10% fetal bovine serum (Invitrogen), 50 U/mL penicillin/streptomycin and 125 ng/mL amphotericin B (GibcoBRL), at 37°C under 5% CO2. The cells were grown onto coverslips in 6-well plates to 30–50% confluence on the day of transfection. Myc-hCG1 and Vpr-GFP (wild-type or mutated) expression vectors were co-transfected using the calcium phosphate method. Briefly, 4 μg of both plasmids were diluted in 60 μL of a 0.24 M CaCl2 solution. The CaCl2-DNA mix was slowly added dropwise to 60 μL of 2 × HBS buffer (50 mM HEPES pH 7.1, 1.5 mM Na2HPO4, 0.28 M NaCl). After 20 min at room temperature, DNA-containing precipitates were slowly dropped onto the surface of the cell culture medium. Cells were incubated at 37°C with 5% CO2 for 6 h, rinsed once with PBS and fresh medium was added before returning to the incubator until analysis. CD4-positive human T cells (HPB-ALL cell line) were kindly provided by G. Bismuth (Institut Cochin, Paris, France), and were maintained in RPMI 1640 medium with Glutamax-1 (Invitrogen) supplemented with 10% fetal bovine serum, 10 mM HEPES buffer, 50 U/mL penicillin/streptomycin and 125 ng/mL amphotericin B (GibcoBRL) at 37°C under 5% CO2. For transient transfections, 1 × 10^7 ^HPB-ALL cells were electroporated as described [59] with 4 μg of a GFP expression vector as a transfection marker, and 16 μg of HA-tagged Vpr (wild-type or mutated) expression vectors. For localization analysis in MDMs, PBMCs were isolated from buffy coats of healthy donors (Etablissement Français du Sang Ile-de-France, Site Saint Vincent-de-Paul) and derived into macrophages for 5 days in complete culture medium [MEM with non essential amino acids (Invitrogen/Gibco) supplemented with 10% FCS, 100 μg/ml streptomycin/penicillin, 1 mM sodium pyruvate, 2 mM L-glutamin and 10 ng/ml rhM-CSF (R&D systems)]. Cells were transfected with the Nucleofector II device (Amaxa GmbH Europe/World), and nucleofection was performed with the Human Macrophage Nucleofector Kit according to the manufacturer's recommendations. Briefly, 6 × 10^5 ^macrophages were nucleofected with 4 μg of plasmid and then cultured in Macrophage-SFM medium (Invitrogen/Gibco) supplemented with 10% FCS and 2 mM L-glutamine for 6 hours. The HEK-293T cells used in the virion packaging assay were maintained in DMEM supplemented with 10% fetal bovine serum, 50 U/mL penicillin/streptomycin and 125 ng/mL amphotericin B (GibcoBRL), at 37°C under 5% CO2. Cells were then co-transfected as described [12], using the calcium phosphate method with 10 μg of the pCMVΔ8.3 proviral vector [60] and 5 μg of wild-type or mutated HA-Vpr expression vector.

### Immunofluorescence staining

18 h after transfection, HeLa cells grown onto coverslips were fixed with 4% paraformaldehyde (PFA) for 20 min and permeabilized with 0.1% Triton X-100 for 10 min. Alternatively, cells were permeabilized for 5 min at 4°C with 55 μg/ml digitonin (Sigma) in transport buffer [61] and then fixed with 4% PFA. Monoclonal antibody to the Myc tag (9E10, Roche) was applied for 30 min followed by a 30-min incubation with Texas Red-conjugated donkey anti-mouse IgG (Jackson). Cells were mounted in PBS containing 50% glycerol. Images were acquired with a Leica DMRB epifluorescence microscope equipped with a CCD camera (Princeton) controlled by Metamorph V5.0r6 software. Optical sections were done using Adobe Photoshop software.

### Cell cycle and apoptosis analysis

Two days after transfection, half of HPB-ALL T cells were collected, rinsed once with PBS and fixed in 1% PFA for 20 min. After two washes in PBS, cells were permeabilized in cold 70% ethanol for 1 h at 4°C. Finally, cells were washed once with PBS, resuspended in PBS containing 200 μg/ml RNase A and 50 μg/ml propidium iodide, and incubated for 15 min at room temperature prior to analysis of DNA content by flow cytometry as described [62]. Three days after transfection, the remaining HPB-ALL cells were rinsed in PBS and analyzed by flow cytometry for exposure of phosphatidylserines (PS) at the cell surface, as an early marker of apoptosis, using phycoerythrin-conjugated annexin V (Annexin V-PE, Bender MedSystems) as described [59]. Cell cycle and PS-exposure profiles were analyzed on a minimum of 5,000 GFP-positive cells using a Cytomics FC 500 instrument (Beckman Coulter).

### Assay for incorporation of Vpr into HIV-1 particles

Incorporation of the Vpr variants was first analyzed using a packaging assay in which HA-tagged Vpr was expressed in *trans *and incorporated into virions [32]. 293T cells were co-transfected with 10 μg of the HIV-1-based packaging vectors pCMVΔR8.3 lacking the *vpr *auxiliary gene, 5 μg of pAS1B-Vpr (wt or mutated) using the calcium phosphate procedure. Cell culture supernatants were harvested 48 h after transfection and filtered through 0.45-μm-poresize filters. Virions were collected by ultracentrifugation for 1.5 h at 120,000 × g at 4°C and suspended in ice-cold lysis buffer containing 1% NP40, 0.5% sodium deoxycholate, 0.05% SDS in PBS and an antiprotease mixture (Roche). For preparation of cell lysates, cells were trypsinized, collected by centrifugation and suspended in ice-cold lysis buffer and clarified by centrifugation. Protein samples from cell and virion lysates were separated by SDS-PAGE and analyzed by Western blotting as previously described [32], using a rat monoclonal anti-HA (3F10, Roche), a mouse monoclonal anti-tubulin (DM1A, Sigma) and a mouse anti-CAp24 (provided from the NIH AIDS Research and Reference Reagent Program).

### Preparation of monocyte-derived macrophages (MDMs)

Peripheral blood mononuclear cells (PBMCs) were isolated from buffy coats of healthy seronegative donors (Centre de Transfusion Sanguine Ile-de-France, Rungis and Hôpital de la Pitié-Salpêtrière, Paris, France), using lymphocyte separation medium (PAA laboratories GmbH, Haidmannweg) density gradient centrifugation. Monocytes were isolated from PBMC by plastic adherence as previously described [63], and non adherent cells (PBLs) were collected, frozen in heat-inactivated fetal calf serum 10% DMSO and stored at -80°C. Monocytes were differentiated in macrophages by culturing for 7–11 days in MDM medium (RPMI 1640 medium supplemented with 200 mM L-glutamine, 100 U penicillin, 100 μg streptomycin, 10 mM HEPES, 10 mM sodium pyruvate, 50 μM β-mercaptoethanol, 1% minimum essential medium vitamins, and 1% nonessential amino acids) supplemented with 15% of human AB serum in hydrophobic Teflon dishes (Lumox™ D Dutcher, Brumath, France). MDMs were then harvested, washed and resuspended in MDM medium containing 10% fetal calf serum. The purity of CD14^+ ^macrophages was usually more than 95% as assessed by immunofluorescence staining and flow cytometry analysis (not shown). Three days before infection, PBLs were thawed and cultured in PBL medium (RPMI 1640 medium supplemented with 200 mM L-glutamine, 100 U penicillin, 100 μg streptomycin, 10% FCS, 100 U/ml of interleukin 2 (Proleukin, Chiron, France) and activated 3 days in presence of 1 μg/ml of PHA (Sigma).

### Virus production and infection

Viruses were produced by transfection of 293T cells with the wild type or mutated YU-2 HIV-1 molecular clones [19] using SuperFect (Qiagen GmbH, Hilden, Germany). Viral supernatants were harvested 72 h after transfection and stored at -80°C. Viral supernatants were centrifuged in Vivaspin centrifugal concentrators (MW cut off 100 000 kDa) against medium to eliminate free p24Gag and cytokines, and then normalized for viral-bound p24 before analysis of Vpr incorporation into virus particles by SDS-PAGE and Western blotting, using a rabbit polyclonal anti-Vpr (provided from the NIH AIDS Research and Reference Reagent Program) and the mouse anti-CAp24. MDMs (0.8 × 10^5 ^cells/well in 96 well plates) and PHA-activated PBLs (10^5 ^cells/well in 96 well plates) were then infected in triplicate with 0.5 ng of p24 using a spinoculation protocol (1 h centrifugation at room temperature at 1,200 × g followed by 1 h incubation at 37°C). Cells were then washed with PBS and cultured in MDM or PBL medium. The supernatant of each well was harvested every 3 or 4 days and fresh medium was added. p24 levels in viral stocks and in infected culture supernatants were measured using a commercial ELISA kit (Beckman Coulter, Paris, France).

## Competing interests

The author(s) declare that they have no competing interests.

## Authors' contributions

GJ, ELR, AD, JM and SBO performed the experimental work. GJ, ELR, AD, FN, GP and SB conceived the experimental strategies and designed individual experiments. SBO, GF and SB analyzed the data and GJ and SB wrote the manuscript. All authors read and approved the final manuscript.
